# Photorealistic Reconstruction of Visual Texture From EEG Signals

**DOI:** 10.3389/fncom.2021.754587

**Published:** 2021-11-19

**Authors:** Suguru Wakita, Taiki Orima, Isamu Motoyoshi

**Affiliations:** ^1^Department of Life Sciences, The University of Tokyo, Tokyo, Japan; ^2^Japan Society for the Promotion of Science, Tokyo, Japan

**Keywords:** visual texture, multimodal variational auto encoder (MVAE), DNN (deep neural network), brain decoding, EEG

## Abstract

Recent advances in brain decoding have made it possible to classify image categories based on neural activity. Increasing numbers of studies have further attempted to reconstruct the image itself. However, because images of objects and scenes inherently involve spatial layout information, the reconstruction usually requires retinotopically organized neural data with high spatial resolution, such as fMRI signals. In contrast, spatial layout does not matter in the perception of “texture,” which is known to be represented as spatially global image statistics in the visual cortex. This property of “texture” enables us to reconstruct the perceived image from EEG signals, which have a low spatial resolution. Here, we propose an MVAE-based approach for reconstructing texture images from visual evoked potentials measured from observers viewing natural textures such as the textures of various surfaces and object ensembles. This approach allowed us to reconstruct images that perceptually resemble the original textures with a photographic appearance. The present approach can be used as a method for decoding the highly detailed “impression” of sensory stimuli from brain activity.

## Introduction

In the field of neuroscience, an increasing number of studies have been conducted to estimate perceptual content and psychological states by extracting certain statistical patterns from brain activity data (Kamitani and Tong, 2005; Schwartz et al., 2006; Miyawaki et al., 2008; Carlson et al., 2011; Green and Kalaska, 2011; Nishimoto et al., 2011). A number of “brain decoding” techniques that identify the object category of an image from the fMRI-BOLD signal have been reported (Shenoy and Tan, 2008; Das et al., 2010; Wang et al., 2012; Carlson et al., 2013; Stewart et al., 2014; Kaneshiro et al., 2015). In recent years, ambitious attempts have been made to reconstruct the image itself from brain activity (Palazzo et al., 2017; Shen et al., 2019a,b). For instance, Shen et al. (Shen et al., 2019a) proposed a method of decoding visual features for each hierarchical stage of visual information processing from an fMRI signal using a deep neural network (DNN) (Krizhevsky et al., 2012; Simonyan and Zisserman, 2015; He et al., 2016) and successfully reconstructed not only the presented image but also the image that an observer imagined in her/his mind.

While excellent decoding is supported by the big data of fMRI, the scope of application is limited by the high costs and potential invasiveness of fMRI. To overcome this limitation, several studies adopted EEG, which provides an easy, cheap, and non-invasive way to collect brain activity data. Palazzo et al. (2017) introduced a method for reconstructing the image of an object from EEG signals by converting the EEG signals into features and conditioning generative adversarial networks (GANs) (Goodfellow et al., 2014) by it. This approach allowed them to reconstruct an image that can be correctly classified into the original object category [EEG classification accuracy: 84%, Inception Score (IS): 5.07, Inception Classification accuracy (IC): 0.43]. However, as pointed out by the authors themselves, their result is a product of a generative model conditioned by categorical information extracted from an EEG signal and not the direct reconstruction of the image itself actually given to the observer. It is evident that this method fails to reproduce aspects of the perceptual realism of an image, such as the detailed shape, sharp contours, and textures. This limitation seems unavoidable considering the small data size of EEG signals, especially in terms of spatial resolution.

Against the above background, it is of interest to explore the use of texture images in decoding from EEG signals. The perception of a texture is based on spatially global image statistics (Julesz, 1965; Heeger and Bergen, 1995; Portilla and Simoncelli, 2000; Landy and Graham, 2004; Freeman and Simoncelli, 2011), and it is even possible to synthesize perceptually similar texture images using only those statistics (Portilla and Simoncelli, 2000). Such statistical information is represented in the low- and mid-level visual cortex, such as V1, V2, and V4 (Freeman et al., 2013; Okazawa et al., 2015, 2017; Ziemba et al., 2019), and used in the rapid perception of scenes, objects, and surface materials (Thorpe et al., 1996; Oliva and Torralba, 2001; Motoyoshi et al., 2007; Rosenholtz et al., 2012; Whitney et al., 2014). In convolutional neural network (CNN), which computationally mimics neural processing in the ventral stream of the visual brain, the spatially global information obtained by the Gram matrix transformation of features extracted from each hierarchical layer stage corresponds to texture representation (Gatys et al., 2015, 2016).

According to these findings, it is expected that texture can be reconstructed from EEG signals by estimating the information that correlates with the spatially global statistics for texture representation. In fact, the recent study (Orima and Motoyoshi, 2021) were able to estimate lower-order image statistics from visual evoked potentials (VEPs) using a linear regression model and synthesize the texture images with identical image statistics. Using the Image-VEP dataset collected in that study, the present paper proposes a CNN-based method that allows a high quality of reconstruction of the original texture image from a VEP for a variety of natural textures.

## Materials and Methods

Texture perception is essentially based on the visual appearance, or impression, of an image according to the continuous perceptual similarity, rather than categorical conceptual knowledge as required for object recognition. From this view, we specifically adopted an MVAE-based approach (Suzuki et al., 2017; Wu and Goodman, 2018; Kurle et al., 2019; Shi et al., 2019; Tsai et al., 2019) that acquires a continuous latent representation shared by a texture image and EEG signal. Using the trained MVAE model, we attempted to reconstruct the texture image from the latent variables obtained when only one-modality information, EEG data, was input.

In our approach, the MVAE model is trained with the texture images and VEP as two-modality information. After training, the latent space shared by the two modalities is acquired in the model. Finally, the test texture image is reconstructed from the latent variable obtained from the corresponding EEG signals input to the trained model.

### EEG Measurement

**FIGURE 1:**
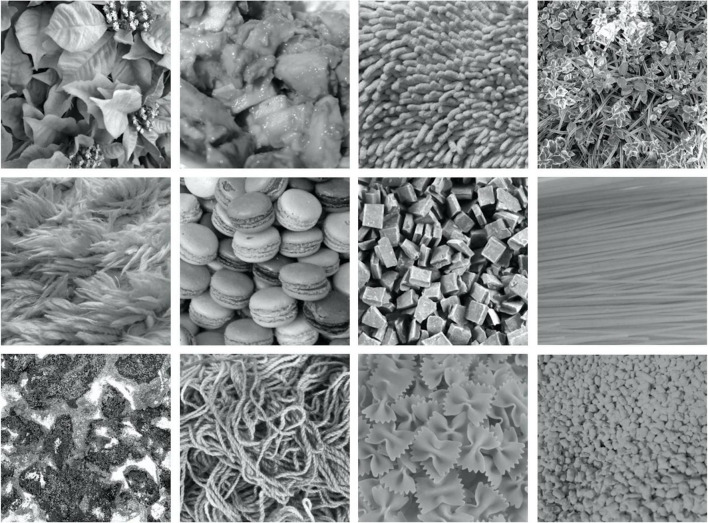
Examples of texture images used in the EEG measurement.

In training the model, we used the dataset obtained by Orima and Motoyoshi (2021). The dataset comprises EEG signals for 166 natural texture images, with each signal measured for a period of 500 ms, 24 times, for each of 15 human observers. Figure 1 shows examples of texture images used in EEG measurements.

Visual stimuli were images of 166 natural textures subtending 5.7 deg × 5.7 deg (256 × 256 pixels). The images were collected from the Internet and our own image database. Each image was achromatic and had a mean luminance of 33 cd/m^2^. In each of 24 measurement blocks, 166 images were presented in random order for 500 ms followed by a 750-ms blank that is equal to a uniform gray background and 15 observers viewed each image with their eyes steadily fixed at the center of the image. During each block, the VEP was measured using 19 electrodes (Fp1, Fp2, F3, F4, C3, C4, P3, P4, O1, O2, F7, F8, T7, T8, P7, P8, Fz, Cz, and Pz according to the international 10/20 method; BrainVision Recorder, BrainAmp Amplifier, EasyCap; Brain Products GmbH) at a 1,000-Hz sampling rate. All stimuli were presented on a gamma-corrected LCD (BENQ XL2420T). The refresh rate of the LCD was 60 Hz, and the spatial resolution was 1.34 min/pixel at an observation distance of 100 cm. All measurements were conducted in accordance with the Ethics Committee for Experiments on Humans at the Graduate School of Arts and Sciences, The University of Tokyo. The participants completed a written consent form.

### Multimodal Variational Auto Encoder for Image Reconstruction From EEG Signals

Considering the continuous and variegated nature of natural textures as visual information, we consider a variational auto encoder (VAE) -based (Kingma and Welling, 2013) approach in which the texture images and the corresponding EEG signals are represented in a continuous latent space.

The VAE is a deep generative model that conducts its generation process by deep learning assuming the existence of a latent variable *z* when data *v* are observed (Kingma and Welling, 2013; Kingma et al., 2014; Dai et al., 2015; Krishnan et al., 2015). Here, by assuming that latent variables are represented on a probabilistic distribution space, we can perform continuous representation learning on the observed input data (Equation 1).


(1) $$\begin{array}{c} z∼p\left(z\right)=N\left(0,I\right),v∼p_{\theta }\left(v|z\right) \end{array}$$

In the VAE, the observed input data *v* are transformed by the encoder into a contractive intermediate representation called latent variable *z*, and the decoder reconstructs the original input data *v*′ with this latent variable as input. The entire model is trained so as to minimize the difference between the input data *v* and the reconstructed data *v*′, and the model parameters of the encoder and decoder are updated. The encoder and decoder comprise a neural network. [In the following, Φ and θrefer to the model parameters of the encoder and decoder, respectively, and the multivariate Gaussian distribution is denoted p(z)].

More practically, the target of training is to maximize the marginal likelihood *p*_θ_(*v*), but because this cannot be treated directly, we optimize the model parameters of the encoder *q*_ϕ_(*z*|*v*)and decoder *p*_θ_(*v*|*z*)to maximize the evidence lower bound (ELBO) given in Equation 2.

In Equation 2, the first term on the right-hand side is called the regularization term. This term regularizes the latent variable z, which is obtained from the mean vector μ and variance vector σ output by the encoder, to distribute according to prior *p*(*z*).

The second term on the right-hand side is the reconstruction error term, which minimizes the difference between the original input data *v* and *v*′, the input data reconstructed from the decoder using the latent variable z. β and λ are weight parameters.


(2) $$\begin{array}{c} E\,L\,B\,O\,\left(v\right)=-\beta \,D_{K\,L}\,\left(q_{\phi }\,\left(z|v\right)|p\,\left(z\right)\right)+E_{q_{\phi }\left(z|v\right)}\,\left[\lambda \,\text{log}\,p_{\theta }\,\left(v|z\right)\right] \end{array}$$

As an extension of the VAE, the multimodal VAE, which treats multimodal information as input, has been proposed (Suzuki et al., 2017; Wu and Goodman, 2018; Kurle et al., 2019; Shi et al., 2019; Tsai et al., 2019). This extension is inspired by the fact that our cognition in the real world uses multimodal information, not unimodal information (Ngiam et al., 2011; Srivastava and Salakhutdinov, 2012; Kiros et al., 2014; Pandey and Dukkipati, 2016). In fact, it is generally known that learning with multimodal information induces the acquisition of better informative representations compared with the case of unimodal information (Ngiam et al., 2011; Srivastava and Salakhutdinov, 2012).

In this study, we apply the extended method for the MVAE (Wu and Goodman, 2018), which allows inference of latent variables even under the partial observation of multimodal information aiming at reconstructing texture images only from EEG signals.

**FIGURE 2:**
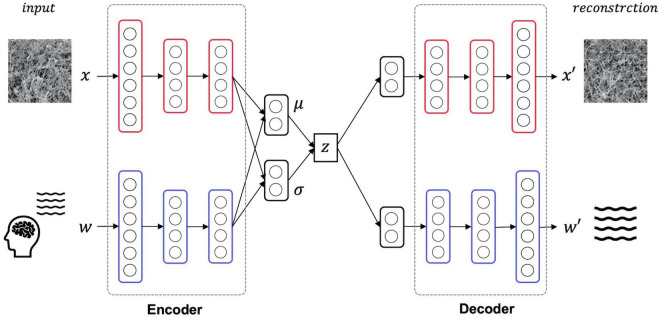
Model overview. The MVAE model comprises an encoder and decoder for the EEG signal modality and an encoder and decoder for the texture stimulus modality. After training this model, the latent space shared by the two modalities is acquired. Thus, the texture stimulus that was presented in the EEG measurement can be reconstructed from the latent variable obtained by inputting only the EEG signals to this trained model.

Here, the texture images and EEG signals are treated as different information modalities, and the latent representation shared by these two modalities is acquired by the learning MVAE. As a result of this training, the stimulus can be reconstructed by decoding the texture image using latent variables acquired by the input of a single modality, the EEG signal. Figure 2 is an overview of the structure of the MVAE model. The MVAE model comprises an encoder and decoder for the EEG signal modality and an encoder and decoder for the texture image modality. By inputting one or both modalities of information into the encoder corresponding to the respective modal information, mean vector μ and variance vector σ can be inferred for the Gaussian distribution. The mean vector μ and variance vector σ obtained here are integrated into, and represented as, a single latent variable using the product of experts (PoE) (Hinton, 2002). Finally, the reconstructed results of EEG signals and texture images are obtained by inputting this latent variable to each of the decoders corresponding to each modal information.

In the training of the MVAE model, there are three possible patterns for the combination of the observable modal information (Here, we denote the texture image modal information as *x*, the EEG signal modal information as *w*, and the observed whole or partial modal information as *V* = {*v*:*v*_1_,*v*_2_,…*v*_*n*_}}.)

• *V*_1_ = {*v*:*x*,*w*}: Both modalities, the EEG signal and texture image, can be observed.
• *V*_2_ = {*v*:*x*}: One modality, the texture image, can be observed.
• *V*_3_ = {*v*:*w*}: One modality, the EEG signal, can be observed.

In reconstructing texture images from EEG signals, which is the target of this study, it is necessary to obtain a representation in the latent space shared by the two modalities of texture images and EEG signals, and to be able to extract latent variables under the partial modal observation (*V_2_*, *V_3_*) that are as good as or similar to those extracted under the full modal observation (*V_1_*). Considering this point, we maximize the ELBO expressed in Equation 3, proposed by Wu and Goodman (2018), in our training.


(3) $$E\,L\,B\,O\,\left(V\right)=-\beta \,D_{K\,L}\,\left(q_{\phi }\,\left(z|V\right)|p\,\left(z\right)\right)+E_{q_{\phi }\left(z|V\right)}[\sum_{v_{i}\in V}\lambda_{i}\,\text{log}\,p_{\theta }\,\left(v_{i}|z\right)]$$

The loss function of the entire model is thus expressed by Equation 4, and the training proceeds accordingly.


(4) $$\begin{array}{c} l\,o\,s\,s=\sum_{V_{i}\in \left\{V_{1},V_{2},V_{3}\right\}}-E\,L\,B\,O\,\left(V_{i}\right) \end{array}$$

One issue that should be considered here is that the image reconstructed using the VAE-based approach is generally blurred. When we tested the reconstruction with the simple VAE using texture images, we found that the reconstruction of fine texture components did not work well, resulting in grayish or blurred images. This is a crucial issue in the present study because we are aiming to realize texture reconstruction with visual similarity to the texture stimuli presented to the observers during EEG measurement. As a solution to such problems, a method combining a VAE and GAN (Rosca et al., 2017) has been proposed to generate natural images and general object images more realistically. However, in the present paper, it is necessary to devise a loss function that improves the reproduction for such texture components when we consider that we use natural texture images in the present study and particularly when failing to reconstruct fine and relatively high spatial frequency components. We thus considered applying precedent knowledge gained in the field of neural style transfer (Gatys et al., 2016; Johnson et al., 2016; Ulyanov et al., 2016; Huang and Belongie, 2017), where texture synthesis is conducted using trained DNN (Gatys et al., 2015).

In general, VGG is used in the implementation of neural style transfer. VGG is a representative DNN that achieved excellent performance in the ILSVRC 2014 (ImageNet Large Scale Visual Recognition Challenge), showing that deepening the layers of the CNN contributes to improved classification accuracy in object recognition tasks (Simonyan and Zisserman, 2015). According to the knowledge in this field, in the trained VGG-19 model (Simonyan and Zisserman, 2015), style information at different levels of abstraction is processed at each stage of the hierarchical processing, and we can extract fine style information at the lower layers and global style information at the higher layers. Additionally, this style information is sufficient for accurate style transferring and texture synthesis (Gatys et al., 2015, 2016; Johnson et al., 2016; Ulyanov et al., 2016; Huang and Belongie, 2017). We therefore use this style information in our approach for more precise texture reconstruction. Specifically, we replace the reconstruction error term in the ELBO with a combination of original reconstruction error term and style error term, which is commonly used in the framework of neural style transfer. The style error is expressed in Equation 5. Here, we denote the input image as *x*, reconstructed image as *x*′, and set of layers in the trained VGG-19 from which the style information can be extracted as L = {1, 2,…,k}. Style information obtained by Gram matrix transformation of the output from each layer is denoted G_*x*_ = {G^*L*^ = 1,G^2^,…,G^*k*^} and ${\hat{\text{G}}}_{x^{{}^{\prime }}}=\left\{{\hat{\text{G}}}^{L=1},{\hat{\text{G}}}^{2},\ldots ,{\hat{\text{G}}}^{k}\right\}$ for the input image and reconstructed image respectively. α is the weighting of style information in each layer, N is the number of filter maps in each layer of VGG-19, and M is the number of elements in each filter map in each layer. *i*, *j* denote the index of the vectorized feature map in layer *l*.


(5) $$\begin{array}{c} \mathrm{StyleLoss}\,\left(x,x^{{}^{\prime }}\right)=\sum_{l=1}^{\text{L}}\alpha_{l}\,\frac{1}{4\,N_{l}^{2}\,M_{l}^{2}}\,\sum_{i,j}{\left({\text{G}}_{i\,j}^{l}-{\hat{\text{G}}}_{i\,j}^{l}\right)}^{2} \end{array}$$

Applying this style error for the loss function confirmed that the texture pattern can be reconstructed clearly regardless of the spatial frequency of the texture in the input image.

### Psychophysical Experiment Setup

In validating the reconstruction results, we carried out a behavioral experiment to examine the relative perceptual similarity of the reconstructed texture to the original. In our display, the original natural texture (2.6 × 2.6 deg, 128 × 128 pixels) was presented at the center, and reconstructed textures were presented on the left and right, 3.5 deg from the center. One reconstructed texture was the target image reconstructed from EEG signals for the central original texture and the other was the non-target image reconstructed from EEG signals for another texture that was chosen randomly from the remaining 165 textures. Six observers with normal or corrected-to-normal vision viewed the stimuli with a free gaze and indicated the texture image (left/right) that was perceptually more similar to the central original texture. Observers were strongly instructed to evaluate the similarity in terms of the visual appearance and not in terms of the categorical meaning. This evaluation was performed on texture images reconstructed using each model trained with the stratified k-fold cross validation (k = 10). For each observer, at least five data for each of the texture images were collected and the probability for each texture image of a response that “the target appeared more similar” was calculated. All experiments were conducted using gamma-corrected LCDs with a refresh rate of 60 Hz (BenQ2720T, SONY PVM-A250, BENQ XL2730Z, BENQ XL2730Z, BENQ XL2730Z, and BENQ XL2735B), each of which was installed in a dark room of the individual observer’s home owing to the COVID19 situation. The viewing distance was adjusted so that the spatial resolution was 1.0 min/pixel. Other parameters were the same as those in the EEG measurements.

## Results

### Multimodal Variational Auto Encoder Model and Training

The MVAE model was trained using a dataset consisting of 166 natural texture images and EEG signals for those images obtained from Orima and Motoyoshi (2021). The dataset was divided into 10 partitions, and stratified k-fold cross-validation (*k* = 10) was performed. Each partition contained the EEG signals for each of the 166 texture images. For evaluation, we conducted psychological experiments using texture images reconstructed by inputting the test data set into the models trained in each cross-validation. In this experiment, we quantitatively evaluated whether the reconstructed image had a high visual similarity to the original image according to the human eye. Measurements of the EEG signals, which were made every 1 ms for 500 ms after the stimulus onset when the texture stimulus was presented to the observer, were taken as the values of 500-dimensional vector data. When we input the EEG signals to the MVAE model, 25–30 samples of EEG signals corresponding to one particular texture stimuli were selected in random combinations and their average waveforms were obtained. Then, for each average waveform, we normalized the maximum value to be 1 and the minimum value to be zero. Among the electrode channels used in the EEG measurement, the signals measured at Fp1, Fp2, F3, F4, C3, C4, P3, P4, O1, O2, F7, F8, T7, T8, P7, and P8 in the international 10/20 method were used as input. Additionally, texture images, as the other information modality, was resized to 128 × 128 on input. At this time, the reconstructed texture image was also output as a 128 × 128 image. In the training, the Adam gradient descent method was used with a learning rate of 1e-4. The batch size was 16. The vector size for the latent variable of the MVAE model was 256. The MVAE model comprises an encoder and decoder that treat the texture images as modal information and an encoder and decoder that treat the EEG signals as modal information. We used 2D-convolution for the encoder and decoder that treat the texture images as modal information, and 1D-convolution for the encoder and decoder that treat the EEG data as modal information. The architectural details of the MVAE model are given in Table 1. In the table, Conv{n}d and UpConv{n}d denote the convolution layer and transposition convolution layer, respectively. n refers to the dimension. The parameter of the convolution (Conv, UpConv) layer is denoted by “Up/Conv{n}d- {kernel size}- {number of channels}-{stride}.” AvgPool refers to average pooling. FC refers to a fully connected layer, and the parameter is FC- {size of each input sample}. ResNetBlock is a convolutional module that can be applied to Reflection padding (where the size of the padding is 1), Convolution, Batch Normalization, and ReLU processes are conducted twice. The parameters of ResNetBlock are given as “ResNetBlock- {kernel size}- {number of channels}-{stride}.” In actual implementation, except for the final output layer, each convolution layer is followed by batch normalization and ReLU rectifier processing in order.

**TABLE 1:** Details of the model architecture.

Texture-image modal		EEG brain signal modal	
	
Encoder	Decoder	Encoder	Decoder
Input 1 × 128 × 128	UpConv2d-3-256-1	Input 16 × 500	FC-256
Conv2d-1-32-1	UpConv2d-2-128-1	Conv1d-3-128-2	UpConv1d-3-256-1
ResNetBlock-3-32-1	ResNetBlock-3-128-1	Conv1d-1-128-1	UpConv1d-4-128-2
AvgPool2d	UpConv2d-4-128-1	Conv1d-1-128-1	UpConv1d-4-128-2
Conv2d-1-64-1	ResNetBlock-3-64-1	Conv1d-1-128-1	UpConv1d-4-64-2
ResNetBlock-3-64-1	UpConv2d-4-64-1	Conv1d-3-256-2	UpConv1d-4-64-2
Conv2d-1-128-1	ResNetBlock-3-32-1	Conv1d-1-256-1	UpConv1d-4-32-2
ResNetBlock-3-128-1	UpConv2d-1-32-1	Conv1d-1-256-1	UpConv1d-4-32-2
Conv2d-1-256-1	(Sigmoid)	Conv1d-1-256-1	UpConv1d-4-1-2
FC-256 (mean), FC-256(var)	Output 1 × 128 × 128	Conv1d-3-512-2	FC-638
		Conv1d-1-512-1	Output 16 × 500
		Conv1d-1-512-1	
		Conv1d-1-512-1	
		Conv1d-1-1024-1	
		Conv1d-1-256-1	
		GlobalAvgPool1d	
		FC-256 (mean), FC-256(var)	

### Reconstruction of the Texture Image

After training the MVAE model, we reconstructed the texture image using the test EEG signals as input. More specifically, the latent variables were extracted from the encoder that treats the EEG signals as modal information, and the texture images were reconstructed by inputting these latent variables to the other decoder that treats the texture image as modal information.

**FIGURE 3:**
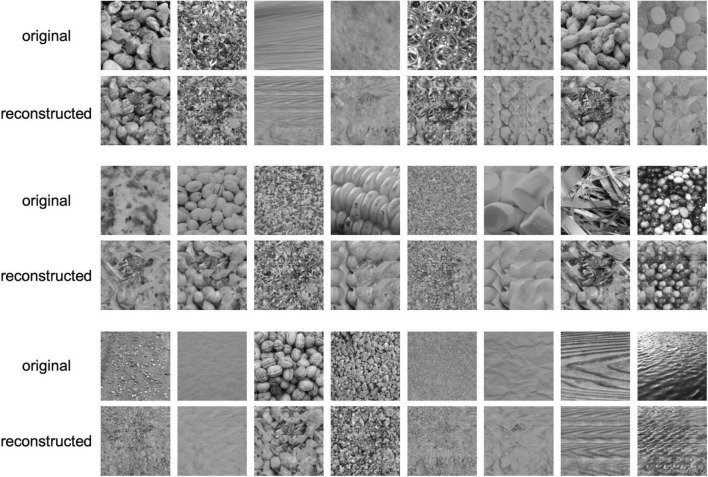
Examples of reconstructed texture images. In each row, the upper images are the original texture images shown to the observer and the lower images are the images reconstructed from EEG.

Figure 3 shows examples of reconstructed images. In each row, the upper images show the original textures, and the lower images show the textures reconstructed from EEG. It is seen that most of the reconstructed textures are remarkably photorealistic, and some are similar to the original textures. The quality of reconstruction is much higher than that of texture synthesis based on linear regression reported in our previous study (Orima and Motoyoshi, 2021).

### Psychophysical Experiment

We conducted the psychological experiment described in section “Psychophysical Experiment Setup” to validate the reconstruction results. Texture images are evaluated in terms of their continuous perceptual appearance, whereas general object images are evaluated based on their categorical semantic classification. Therefore, in this psychological experiment, we instructed the observers to select the reconstructed texture image that is closer to the original image in pure visual appearance without being confined to the categorical classification.

We prepared 10 samples of reconstructed texture images for each of the 166 different texture images. Each sample for a particular texture image was reconstructed from each of the 10 models in the stratified k-fold cross validation (*k* = 10). Six observers participated in the experiment, and each of the observers followed the experimental procedure five times in evaluating the reconstruction result for each of the 166 textures. After numbering the 10 samples reconstructed from each cross-validation model in order (1, 2, 3,…, 10), we assigned odd-numbered samples to three observers and even-numbered samples to the remaining three observers. Each observer performed 830 trials (166 trials, five sessions), and the total number of trials for all observers was thus 4,980.

The results of the experiment show that the correct identification rate in all trials was 70.1%, which was significantly higher than the chance level (50%) based on the binomial test (*p*≪ 0.001). For all the six individual observers, we found that the correct identification rate for 166 textures was significantly higher than the chance level (50%) in a one-tailed *t*-test (*t*(165) > 9.77,*p* < 4.41*e*−18). Together with the observations presented in Figure 3, these results suggest that the reconstruction was successful.

**FIGURE 4:**
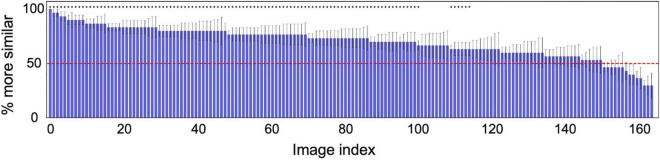
Probability of a response that the image reconstructed from the original texture (target) was more similar than the image reconstructed from another random texture (non-target) to the original image. The horizontal axis shows the index of the texture image, sorted from the left in descending order of the probability. The red line denotes the chance level (50%). The error bars indicate ± 1 s.e.m. across observers. The asterisks indicate that the average identification rate across six observers for that texture is statically significant in a one-tailed *t*-test (*p* < 0.05).

For more analysis, Figure 4 shows the probability of a response that “the target appeared more similar” averaged across the six observers for each of 166 textures. The horizontal axis is the index of the texture image, sorted from the left in descending order of the proportion correct. The horizontal red line denotes the chance level (50%). The asterisks indicate that the average identification rate across the six observers for that texture is statically significant in a one-tailed *t*-test (*p* < 0.05).

While our method has performed with a certain degree of success, the reconstruction is limited to the textures used for training the MVAE model. The establishment of a more versatile reconstruction approach requires the consideration of the possibility of reconstruction for unknown novel textures that were not used in the model training phase. We therefore considered conducting a limited test of reconstruction on unknown novel textures. However, it should be explicitly stated at the outset that the validation in this limited test was not sufficient. The dataset used in the present study was collected in our previous study (Orima and Motoyoshi, 2021), which was not carried out for the purpose of brain decoding. Therefore, the dataset was not adequate for considering unknown novel texture reconstruction methods based on sufficient cross-validation. The results presented below are examined under this constraint.

The novel texture reconstruction based on the MVAE-based approach proposed in this study is expected to be realized using the intermediate representation of other multiple textures on the latent space acquired using a variety of textures according to the nature of the method. Specifically, the acquisition of an internal representation that can represent the new texture is important. This is analogous to the use of visual features in pre-trained DNNs as a proxy for hierarchical visual representation in the brain in visual decoding study with fMRI data (Horikawa and Kamitani, 2017; Shen et al., 2019a). Considering this point, we prepared the dataset in the following manner. We used 140 of the 166 textures for training and 26 for testing in this limited test. In preparing these test textures, we created 83 visually similar pairs from 166 textures based on VGG’s style information. Of these pairs, we manually selected 26 pairs that did not overlap in visual impression between the pairs. One of the textures in each of these 26 pairs was picked as the 26 textures for the test dataset. The setting in model training was the same as that in cross-validation training.

**FIGURE 5:**
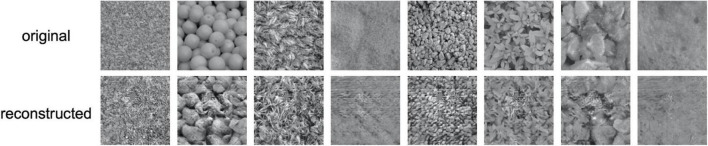
Examples of unknown novel texture reconstruction. The upper images are the original texture images shown to the observer and the lower images are the images reconstructed from EEG.

**FIGURE 6:**
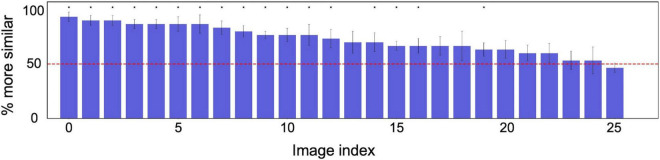
Psychological experimental results on texture images reconstructed from EEG signals corresponding to unknown novel textures in training. The correct identification rate averaged across six observers for 26 unknown novel textures, sorted from the left in descending order of the correct identification rate. The horizontal red line denotes the chance level (50%). The error bars indicate ± 1 s.e.m. across observers. The asterisks indicate that average identification rate across the six observers for that texture is statically significant in a one-tailed *t*-test (*p* < 0.05).

Figure 5 shows examples of reconstructed images on the limited test. The upper images show the original textures, and the lower images show the textures reconstructed from EEG signals. To evaluate the reconstruction results on novel textures, we conducted a psychological experiment with the same procedure as described in section “Psychophysical Experiment Setup.” We prepared five samples of reconstructed texture images for 26 novel texture images in the test dataset. The six observers evaluated the reconstruction results for each of 26 textures (five repetitions for each). We found that the average correct identification rate was 72.8%. For all of six individual observers, the correct identification rate was significantly higher than the chance level (50%) in a one-tailed *t*-test (*t*(25) > 4.18,*p* < 0.0003). Figure 6 shows the correct identification rate averaged across the six observers for 26 unknown novel textures, sorted from the left in descending order of the correct identification rate. The horizontal red line denotes the chance level (50%). The asterisks indicate that the identification rate over the six observers for that texture is statically significant in a one-tailed *t*-test (*p* < 0.05).

## Discussion

The present study introduced a method in which an MVAE is used to reconstruct the image of a natural texture from EEG signals alone. Our trained MVAE model successfully reconstructed the original texture with photorealistic quality and greatly outperformed linear regression on the same dataset (Orima and Motoyoshi, 2021).

As mentioned earlier, it is generally challenging to decode neural representations of a natural scene with EEG because of the low retinotopic resolution of EEG as compared with that of fMRI. The present study avoided this limitation by confining the scope to textures for which the perception is determined by spatially global image statistics, and we successfully reconstructed various natural textures from EEG signals. The previous study having a similar scope (Orima and Motoyoshi, 2021) focused on understanding the neural dynamics for image statistics assumed in human texture perception (e.g., Portilla–Simoncelli statistics) and demonstrated a reconstruction of textures using image statistics linearly regressed from EEG signals. In contrast, the present study pursued a technique to reconstruct an image with higher quality and showed that the use of an MVAE allows the reconstruction of textures with high quality.

The previous approach reconstructed natural object images from EEG signals on the basis of the classification of discrete object categories acquired in a supervised network (Palazzo et al., 2017). In contrast, the present study aimed to reconstruct a purely perceptual impression without any dependency on top-down knowledge such as that of categories, by acquiring a continuous representation space of visual textures in a fully unsupervised learning manner. The resulting images duplicated the perceptual impression well. Of course, such success might be possible only for the textures that we used, and it is unclear if the present approach is applicable to a wide range of classes of images, such as images of objects and scenes. However, we believe that the fact that we were able to reproduce images from EEG signals in a highly realistic manner brings a new direction in the decoding of sensory information. We are currently applying the same approach to sounds.

**FIGURE 7:**
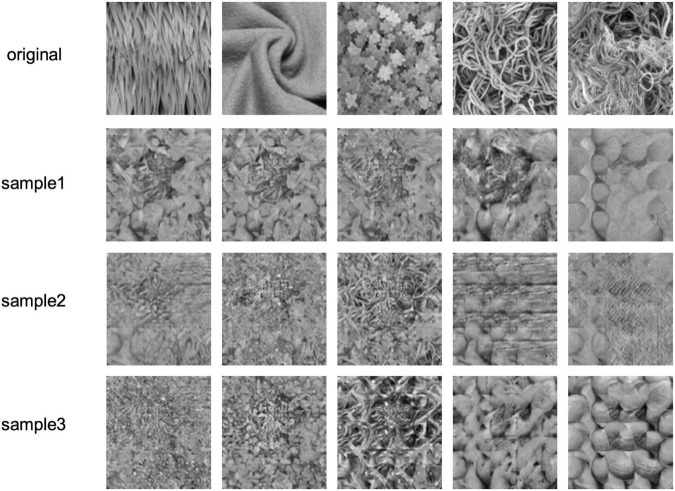
Failure examples of the reconstructed texture images having the lowest “more similar” response rates in the psychophysical experiment. The upper row shows the original textures and the other rows show three sample images reconstructed from EEG signals.

We should also note a limitation of the present approach. Figure 7 shows the worst examples of texture reconstruction. The upper images show the original texture, and the other images show the image samples reconstructed from EEG signals. These reconstructed images are similar to one of the other textures tested, and there is a large variability among samples for the same original texture. This result is due to the VAE acquiring continuity on visual similarity between the considered textures in the latent space, and therefore, when the proper texture representation was not extracted from the EEG signals, the representation became an intermediate representation that was determined virtually randomly in the space defined by the limited number of textures that we used. As a result, it is highly possible that the reconstructed texture is similar to one of the other original textures. This problem could be avoided with a latent space that is richer with more diverse texture images. However, such a latent space would require many more images and corresponding EEG data.

A more generally applicable texture reconstruction from EEG signals requires consideration of the possibility of reconstruction on unknown novel textures in the model training phase. To investigate this point, we performed a limited study on reconstruction for novel textures by dividing the 166 textures into training and test. While there are challenges for certain textures, such as reconstruction being unstable from sample to sample, the result shows the possibility of reconstruction for novel textures within the framework of the proposed MVAE-based approach. However, it should be noted again that because we used a dataset that measured for a different purpose in our previous study (Orima and Motoyoshi, 2021), we could not examine the results based on sufficient cross-validation in this limited test. This problem needs to be investigated in future work with a sufficiently extended dataset.

As the next advancement in the analysis of texture representation on EEG signals, an investigation of the frequency components on EEG signals may provide new findings. The frequency components that contribute to the reconstruction of a certain texture can be identified by evaluating the reconstruction results with and without stripping certain frequency components in texture reconstruction using the MVAE model. This is expected to be valuable in understanding the correspondence between the frequency bands in texture processing in the brain that correspond to each of the various textural representations.

While the present approach provides an effective tool for reconstructing the visual impression of an image with complex spatial structures from EEG signals, there is still room for improvement. In this study, the model was trained using an EEG signal dataset for 166 texture images, which was divided for cross-validation regardless of the observer. Therefore, we were unable to analyze the characteristics of visual impressions for each observer. As a future development, it would be interesting to conduct research focusing on the differences in visual impressions and visual functions specific to a particular individual, although there are still challenges in measuring sufficient data for each observer and methodological challenges in avoiding inadequate data. In the present study, we focused on the pipeline of reconstructing texture stimuli from EEG signals, but owing to the nature of MVAE-based systems, it is also possible to consider the opposite pipeline through which the EEG signal is reconstructed from an image.

## Data Availability Statement

The data supporting the conclusions of this article will be made available by the authors upon reasonable request.

## Ethics Statement

The studies involving human participants were reviewed and approved by the Ethics Committee for Experiments on Humans at the Graduate School of Arts and Sciences, The University of Tokyo. The patients/participants provided their written informed consent to participate in this study.

## Author Contributions

SW, TO, and IM designed the study. TO and IM provided the dataset of images and EEG. SW developed, trained the model for texture reconstruction, performed the psychophysical experiment, and analyzed the data. SW and IM wrote the first draft of the manuscript. All authors contributed to manuscript revision, read, and approved the submitted version.

## Conflict of Interest

The authors declare that the research was conducted in the absence of any commercial or financial relationships that could be construed as a potential conflict of interest.

## Publisher’s Note

All claims expressed in this article are solely those of the authors and do not necessarily represent those of their affiliated organizations, or those of the publisher, the editors and the reviewers. Any product that may be evaluated in this article, or claim that may be made by its manufacturer, is not guaranteed or endorsed by the publisher.
